# Green Tea Polyphenol Catechins Inhibit Coronavirus Replication and Potentiate the Adaptive Immunity and Autophagy-Dependent Protective Mechanism to Improve Acute Lung Injury in Mice

**DOI:** 10.3390/antiox10060928

**Published:** 2021-06-07

**Authors:** Chih-Ching Yang, Chang-Jer Wu, Chen-Yen Chien, Chiang-Ting Chien

**Affiliations:** 1 Department of Life Science, School of Life Science, College of Science, National Taiwan Normal University, Taipei 11677, Taiwan; mdyangcc@mohw.gov.tw; 2 Office of Public Relation of Ministry of Health and Welfare, Taipei 115204, Taiwan; 3 Center for General Education, Mackay College of Medicine, Nursing and Management, New Taipei City 11260, Taiwan; 4 Department of Food Science, Center of Excellence for the Oceans, National Taiwan Ocean University, Keelung 202301, Taiwan; cjwu@mail.ntou.edu.tw; 5 Department of Health and Nutrition Biotechnology, Asia University, Taichung 41354, Taiwan; 6 Graduate Institute of Medicine, Kaohsiung Medical University, Kaohsiung 80708, Taiwan; 7 Mackay Junior College of Medicine, Nursing and Management, New Taipei City 11260, Taiwan; ccywyt@msl.mmh.org.tw

**Keywords:** acute lung injury, adaptive immunity, autophagy, catechins, cytokine storm, SARS-CoV replication

## Abstract

Effective antiviral therapeutics are urgently required to fight severe acute respiratory syndrome (SARS) caused by a SARS coronavirus (SARS-CoV). Because polyphenol catechins could confer antioxidative, anti-inflammatory, antiviral, and antimicrobial activities, we assessed the therapeutic effects of catechins against SARS-CoV replication in Vero E6 cells, the preventive effect of catechins on CD25/CD69/CD94/CD8^+^ cytotoxic T lymphocytes-mediated adaptive immunity, and the protective effect on lipopolysaccharide-induced acute lung injury (ALI) in mice. We found that catechins containing 32.8% epigallocatechin gallate, 15.2% epicatechin gallate, 13.2 epicatechin, 10.8% epigallocatechin, 10.4% gallocatechin, and 4.4% catechin directly inhibited SARS-CoV replication at sub-micromolecular concentrations. Four-week catechins ingestion increased CD8^+^ T cell percentage, upregulated CD69^+^/CD25^+^/CD94-NKG2A/CD8^+^ T lymphocytes-mediated adaptive immunity, and increased type I cytokines release responding to ovalbumin/alum. Catechins significantly reduced lipopolysaccharide-induced cytokine storm and oxidative stress and ALI by inhibiting PI3K/AKT/mTOR signaling to upregulate Beclin-1/Atg5-Atg12/LC3-II-mediated autophagy mechanism. Pretreatment of autophagy inhibitor 3-Methyladenine reversed the inhibiting effects of catechins on the cytokines and oxidative stress levels and ALI. In conclusion, our data indicated that catechins directly inhibited SARS-CoV replication, potentiated the CD25/CD69/CD94/CD8^+^ T lymphocytes-mediated adaptive immunity and attenuated lipopolysaccharide-induced ALI and cytokine storm by PI3K/AKT/mTOR-signaling-mediated autophagy, which may be applied to prevent and/or treat SARS-CoV infection.

## 1. Introduction

A life-threatening severe acute respiratory syndrome (SARS) induced by a coronavirus, SARS-CoV, with relative high morbidity and mortality was first reported in Southern China and Hong Kong [1,2,3,4]. Recently, the outbreak of coronavirus disease 2019 (COVID-19) caused by SARS-CoV-2, along with the lack of targeted medicaments and vaccines, forced the world to find out new antiviral agents. It has spread rapidly to almost all parts of China and many other countries. As of now, the incidence of COVID-19 continues to rise. Cumulated reports evidenced that some patients with severe COVID-19 might display a syndrome with a distinct cytokine storm [5,6], reduction in CD8^+^ cell [7], and acute lung injury (ALI) associated with pulmonary inflammation and increased microvascular permeability [8]. SARS-CoV and SARS-CoV-2 could induce acute respiratory distress syndrome (ARDS), pulmonary inflammation, cytokine storm, and multiple organ failure, which is mimicked by lipopolysaccharide (LPS), a bacterial endotoxin present on the outer membrane of Gram-negative bacteria, evoked ARDS and cytokine storm [9], suggesting SARS-CoV-2 and LPS-induced ALI/ARDS and cytokine storm possibly through some similar pathways. Autophagy is a highly conserved biological process that occurs in eukaryotes and maintains cell homeostasis and viability by recycling and reusing energy through PI3K/AKT/mTOR signaling to regulate the autophagy-related proteins expression, such as Beclin-1/Atg5-Atg12/LC3-II [10]. It has been suggested that host autophagy may function in an antiviral capacity to suppress virus infection [11]. SARS-CoV-2 is RNA virus with a genetic structure similar to that of SARS-CoV or Middle East Respiratory Syndrome (MERS)-CoV. The viral RNA encodes a protease to produce viral proteins and to control the activity of the replicase complex and is necessary for virus replication and infection, making it a perfect target for designing antiviral therapies. Given the urgency of the COVID-19 or SARS treatments, repurposing of old or traditional drugs is an attractive proposition due to the use of de-risked compounds with potentially lower costs and especially shorter development timelines. A candidate drug should meet the requirements to (1) inhibit SARS-CoV replication, (2) enhance CD8^+^ mediated adaptive immunity, (3) depress cytokine storm, and (4) promote autophagy-dependent protective mechanism to ameliorate ALI.

To provide an immediate prevention and cure, we suggested that many plant-derived natural compounds (polyphenols) might provide a starting point for the research on the use of plant extracts in coronavirus treatment and prevention. For the last several years, we have studied green tea (*Camellia sinensis*) extracts polyphenols for their different therapeutic and disease protective roles. Antivirus polyphenolic drugs can inhibit coronavirus enzymes, which are essential for virus replication and infection. Regarding the antiviral role, tea polyphenols have been demonstrated to be strong agents [12]. For example, green tea extract catechins can provide a key to inhibit Hepatitis B virus infection [13], catechin can prevent influenza A (H1N1) virus infection, catechin can interact with SARS-CoV-2 by binding with S protein [14] and epigallocatechin gallate (EGCG) can inhibit human coronavirus replication [15]. In addition, cumulated evidence indicated that the regular consumption of green tea decreases influenza infection and some cold symptoms and may protect from influenza virus [16]. In this study, we aimed to evaluate potential anti-inflammatory and antiviral catechins, as a new strategy for directly inhibiting SARS-CoV replication, promoting CD25/CD69/CD94 expression on CD8^+^ cytotoxic T cells to enhance adaptive immunity and attenuating LPS evoked ALI and cytokine storm through PI3K/AKT/mTOR mediated Beclin-1/Atg5-Atg12/LC3-II related autophagy in mice.

## 2. Methods and Materials

### 2.1. Materials

**Figure 1:**
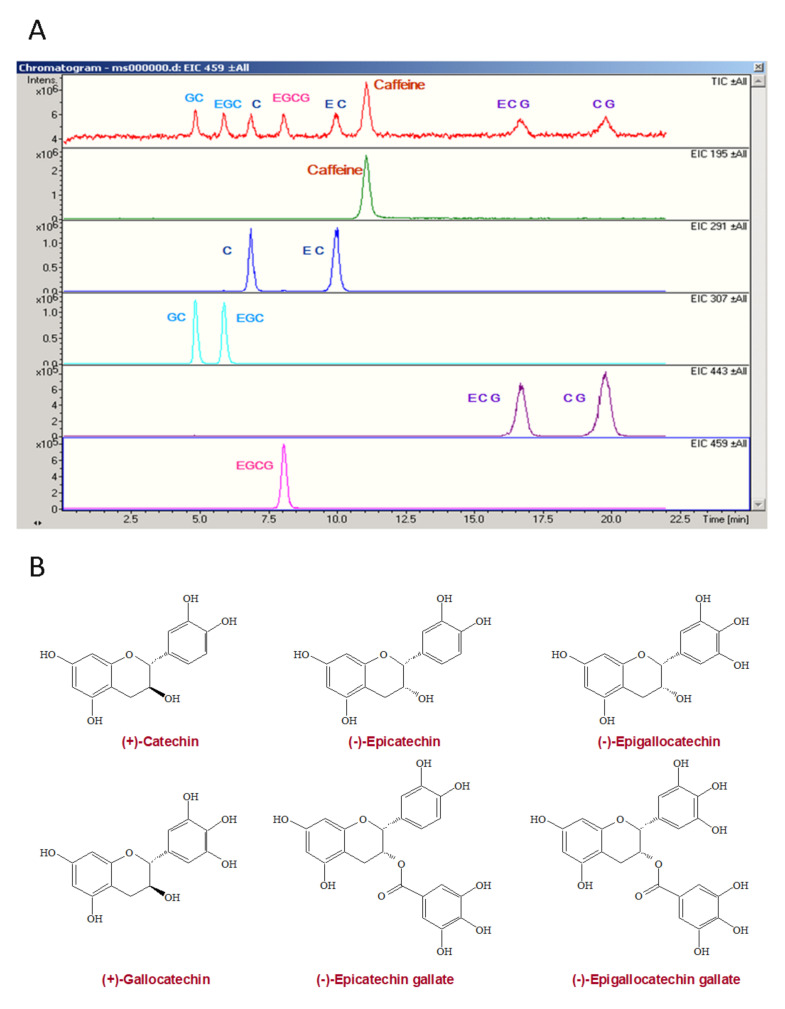
The HPLC analysis of green tea extracts catechins (**A**) and chemical structures of catechins (**B**). C, catechin; EC, epicatechin; GC, gallocatechin; CG, catechin gallate; EGC, epigallocatechin; CAF, caffeine; ECG, epicatechin gallate; EGCG, epigallocatechin gallate.

The decaffeinated green tea extracts (Numen Biotech Co., LTD, Taipei, Taiwan), which contain 328 mg of epigallocatechin gallate, 152 mg of epicatechin gallate, 132 mg of epicatechin, 108 mg of epigallocatechin, 104 mg of gallocatechin, and 44 mg of catechin (Figure 1) in 1 g of green tea extracts were identified by the Esquire3000plus™ LC/MS Ion-Trapsystem (Bruker Daltonik, GmbH, Bremen, Germany) at a flow rate of 0.5 mL/min of mobile phase (25% Methanol, 74.9% distilled water, and 0.1% formic acid (*v*/*v*)) with Eclipse XDB-C_8_ columnratic).

### 2.2. Catechins Effects on SARS-CoV Replication In Vero E6 Cells

This part of study was performed in the P4 laboratory of the Institute of Preventive Medicine of National Defense Medical College (Sanxia District, New Taipei City, Taiwan). The effect of different concentrations of catechins were applied to determine the inhibitory effect on SARS-CoV replication. In this part of study, the Vero E6 cells were infected with Hong Kong strain of SARS-CoV at the multiplicity of infection (MOI) of 0.1. Under the 0.1 MOI level, these cells began to display mild cytopathic effects (CPE) at 1 day post-infection, and the typical CPE as reported by Ksiazek et al. [3] was found at 2 days post-infection. Different concentrations of catechins were applied to the Vero E6 cell culture 1 h before SARS-CoV infection and evaluated for their effects on the degree of inhibition of SARS-CoV replication as measured by the CPE level.

The effect of catechins on SARS-CoV replication was further determined with the immunoblot analysis. Vero E6 cells were maintained in Dulbecco’s modified Eagle’s medium (DMEM) medium supplemented with 10% fetal bovine serum. In each well, 4 × 10^4^ cells were seeded to form a cell layer approximately 70% confluent. During SARS-CoV infection, DMEM medium contained 2% fetal bovine serum. The green tea extracts catechins were dissolved at several concentrations up to 6.25 mg/mL in the medium. The cell lysates were obtained at 48 h post-infection and analyzed by immunoblotting with the antiserum derived from a convalescent SARS patient, which was used as the primary antibody. After the primary antibody stained, the proteins were stained with HRP-conjugated goat anti-human IgG (Jackson ImmunoResearch Laboratories, Inc., West Grove, PA, USA, 1:1000) and detected with an ECL kit system (Amersham Biosciences, Piscataway, NJ, USA).

Alternatively, we adapted an immunofluorescence assay (IFA) to determine the inhibitory effect of catechins on viral antigen synthesis. Catechins were added to cells at serial dilution. After 48 h post-infection, these cells were fixed and stained with anti-serum from the same convalescent SARS patient described above. The cell viability was determined by using MTS [3-(4,5-dimethylthiazol-2-yl)-5-(3-carboxymethoxyphenyl)-2-(4-sulfophenyl)-2H-tetrazolium] assay described previously [17].

To examine whether the virus yield was also reduced by catechins, culture supernatants were collected and RT-PCR was performed to measure viral RNA yields. Two days post-infection, viral RNA was isolated from 140 µL of supernatant of the SARS-CoV infected cells with a QIAamp^®^ viral RNA mini kit (Qiagen^®^, Hilden, Germany) in accordance with the manufacturer’s instructions. Then, 1 µL of extracted viral RNA was applied for RT-PCR by using a Qiagen^®^ One Step RT-PCR kit with SARS-specific primers Cor-p-F2 5′CTAACATGCTTAGGATAATGG3′ and Cor-p-R1 5′CAGGTAAGCGTAAAACTCATC3′. This primer set was designed to amplify a region in open reading frame 1b in the genome of SARS-CoV [3]. The RT-PCR cycles were designed as follows: 50 °C for 30 min; 95 °C for 15 min; 25 cycles of 94 °C for 30 s, 50 °C for 30 s, 72 °C for 1 min, and 72 °C for 10 min.

### 2.3. Animal Studies and Grouping

In the first part of animal study, we used 40 female Balb/c mice with 6–8 weeks purchased from BioLASCO Taiwan Co. Ltd. (I-Lan, Taiwan), housed at the Experimental Animal Center, National Taiwan Normal University, at a constant temperature and with a consistent light cycle (light from 07:00 to 18:00 h), and divided into control, low-dose catechins (25 mg/kg/day) feeding, median-dose catechins feeding (50 mg/kg/day), and high-dose catechins feeding (125 mg/kg/day) for 4 weeks in this study (*n* = 10 each). In the second part of animal study, 40 female Balb/c mice were randomly divided into four groups (10 mice per group), control group, LPS (10 mg/kg) group, catechins (50 mg/kg) + LPS group, and 3-MA (15 mg/kg) + catechins + LPS group. Then, 3-MA was administered 0.5 h before catechins intervention, and catechins intervention was administered every day, for 3 days, before LPS treatment. Equal amounts of PBS were administered to the control group. Each group of mice was sacrificed after 24 h of LPS treatment. All drugs were administered through intraperitoneal injection. The mice were fed standard chow (Laboratory Rodent Diet 5001 from Young Li Trading Co., Ltd., New Taipei City, Taiwan containing 0.4% sodium) and tap water ad libitum. All the surgical and experimental procedures were approved by the ethics committee “Institutional Animal Care and Use Committee of National Taiwan Normal University” (identification code of approval is 108,030, and date of approval is 03/10/2020) and were in accordance with the guidelines of the National Science Council of Republic of China (1997) and ARRIVE guidelines. Because one cup of green tea contains ≈100–150 mg catechins, the total amount of catechins (50–125 mg) ingested was comparable to 0.5–1 cup of green tea [18].

### 2.4. T-Cell Activation

Splenic T lymphocytes obtained from catechins administrated to mice were seeded at 5 × 10^6^ cells/mL with 10 µg/mL concanavalin A (Con A) (Sigma, Saint Louis, MO, USA) for 3 days. Then, dead cells were removed by Ficoll-Paque solution (Pharmacia, Stockholm, Sweden). The purity of T cells in live cells was determined by CD3ε-PerCP staining (Pharmingen) on FACS Calibur (Becton Dickinson, Mountain View, CA, USA) and the purities in control, 25 mg/kg, 50 mg/kg, and 125 mg/kg groups were 93.4%, 95.0%, 92.2%, and 92.6%, sequentially. Purified T cells were then set for total RNA extraction and cytokine expression analysis.

### 2.5. Immunization and Challenge

After 4-week catechins administration, the mice were sensitized and boosted with 10 μg ovalbumin/alum (OVA) (Sigma) via intraperitoneal injection in one week-intervals. Sterile PBS was utilized in the control group. Seven days later, splenocytes isolated from Balb/c mice were challenged with 100 μg/mL OVA or PBS for 2 days. The culture supernatant was collected for cytokine detection and the cells were prepared for RNA extraction.

### 2.6. Real-Time Quantitative PCR

In this assay, cDNA samples were synthesized from total RNA firstly. We used Assay-On-Demand™ gene expression products (Applied Biosystems, Foster City, CA, USA) to detect and quantify the appearance of TNF-alpha (Assay ID: Mm00443258_m1), IFN-γ (Assay ID: Mm00801778_m1), IL-2 (Assay ID: Mm00434256_m1), IL-4 (Assay ID: Mm00445259_m1), IL-5 (Assay ID: Mm00439646_m1), and IL-10 (Assay ID: Mm00439616_m1) in cDNA samples. At the same time, we selected TBP (Assay ID: Mm00446973) as the endogenous control. Briefly, 25 μL 2× TaqMan^®^ Universal PCR Master Mix (Applied Biosystems) were reacted with 2.5 μL 20× Assay-On-Demand™ gene expression assay mix and 2 μL cDNA diluted in 20.5 μL RNase-free water for each 50 μL reaction. The thermal cycling parameters was utilized as follow, 50 °C for 2 min, 95 °C for 10 min and denaturation at 95 °C for 15 s, annealing and extension at 60 °C for 1 min for 40 cycles. The reactions were performed on ABI PRISM^®^ 7700 sequence detector, and the data were analyzed by SDS 1.9.1 software. Each specimen was detected at least in duplicate. 

### 2.7. Cytokine ELISA

Type I cytokines (IL-2 and IFN-γ) and type II cytokines (IL-4 and IL-10) secreted in culture supernatant and plasma IL-2 and IFN-γ were analyzed by R&D^®^ (Minneapolis, MN, USA). ELISA kits and all procedures were carried out as described in the manufactures’ guidelines.

### 2.8. Cytokine Array

The secretion of various cytokines into conditioned media from OVA-stimulated splenocytes was evaluated in six catechins-fed and two control mice, using Mouse Cytokine Antibody Array III obtained from RayBiotech, Inc. (Norcross, GA, USA), according to the manufacturer’s protocol with some modification. This assay can simultaneously detect 62 different cytokines and chemokines with high specificity and sensitivity. Briefly, membranes were incubated with 1× blocking buffer at room temperature for 1 h. After decantation of the blocking buffer, 2.5 mL conditioned supernatant was added to membranes and incubated at 4 °C, overnight. Then the membranes were subjected to three 5-min washes with 1× wash buffer I, followed by two 5-min washes with 1× wash buffer II. After washing, membranes were incubated with biotin-conjugated antibodies at room temperature for 2 h and washed as described previously. The membranes were then incubated with HRP-conjugated streptavidin at 4 °C overnight. Unbound reagents were washed away. The membranes were developed with detection mixture provided in the kit and exposed to X-ray film (Kodak x-omat AR film). To determine the relative concentrations of cytokines in the media, the densities of individual spots were measured by using the Fugifilm Image Gauge V3.46 software. The results were expressed as relative densities compared with blank included in each membrane that was considered as zero.

### 2.9. Catechins Effects on LPS Induced Cytokine Storm and ALI

Cytokine storm is a very commonly observed factor in most severe COVID-19 and SARS patients and is also one of the leading causes of mortality [19]. Severe COVID-19 patients have displayed a high level of cytokine storm [20]. LPS-evoked sepsis induced ALI and cytokine storm may mimic these aspects of COVID-19 and SARS [9]. We thus determined the catechins effect on plasma IL-2 and IFN-γ secretion in the LPS-treated mice.

### 2.10. Bronchoalveolar Lavage Fluid (BALF) and Reactive Oxygen Species (ROS) Analysis

After the experiment, bronchoalveolar lavage (BALF) fluid was obtained by injecting 1 mL of Hanks’ balanced salt solution via the tracheal cannula into the lungs, followed by collecting the fluid. Cells were collected from the BAL fluid by centrifugation (500× *g*, 20 min, 4°C) and counted by using an automated cell counter (TC20; Bio-Rad, Hercules, CA, USA). BALF was determined ROS by a luminol-amplified chemiluminescence method as described previously [18]. After sacrificing the mice, the blood from the vena cava was immediately collected (300× *g* for 5 min). The plasma was used for cytokine analysis.

### 2.11. Histopathology of Mouse Lungs

Mouse lungs were fixed in 4% formalin, embedded in paraffin, sectioned to 5 μm-thick sections, mounted onto slides, and stained with hematoxylin and eosin (H&E).

### 2.12. Immunoblotting of Lung Tissues

We used immunoblotting techniques to measure lung protein levels of the autophagy-related Beclin-1, Atg5-Atg12 and LC3-II/LC3-I, apoptosis-related caspase 3 and poly-(ADP-ribose)-polymerase (PARP), and PI3K/AKT/m-TOR signaling. In brief, proteins (20 μg) were separated on 10% polyacrylamide gels and electrophoretically transferred to nitrocellulose membranes (Amersham Biosciences, Buckingham, England, UK). The membranes were blocked and then incubated overnight, at 4 °C, with antibodies raised against Beclin-1 (Cell Signaling Technology, Inc., Danvers, MA, USA), Atg5-Atg12 (Gene Tex, Alton Parkway, Irvine, CA, USA), and LC3-II/I (Cell Signaling Technology); the activation fragments (32 kDa of proenzyme and 17 kDa of cleaved product) of caspase 3 (CPP32/Yama/Apopain, Upstate Biotechnology, Lake Placid, NY), PARP (Cell Signaling Technology), p-PI3K (Cell Signaling Technology), p-Akt (Cell Signaling Technology), p-mTOR (Cell Signaling Technology), and β-actin (Sigma) were used. The density of the band with the appropriate molecular mass was determined semi-quantitatively by densitometry, using an image-analyzing system (Alpha Innotech, San Leandro, CA, USA).

### 2.13. Statistical Analysis

All values are expressed as means ± standard error mean (SEM). Between-group comparisons were performed by using unpaired *t*-tests or analysis of variance with Bonferroni method as post hoc analysis; within-group comparisons were performed by using paired *t* tests or repeated-measures analysis of variance with Bonferroni method as post hoc analysis. A value of *p* < 0.05 was adapted to indicate statistical significance. All computations were performed by using SPSS for WINDOWS software (version 13.0; SPSS Inc., Chicago, IL, USA).

## 3. Results

**Figure 2:**
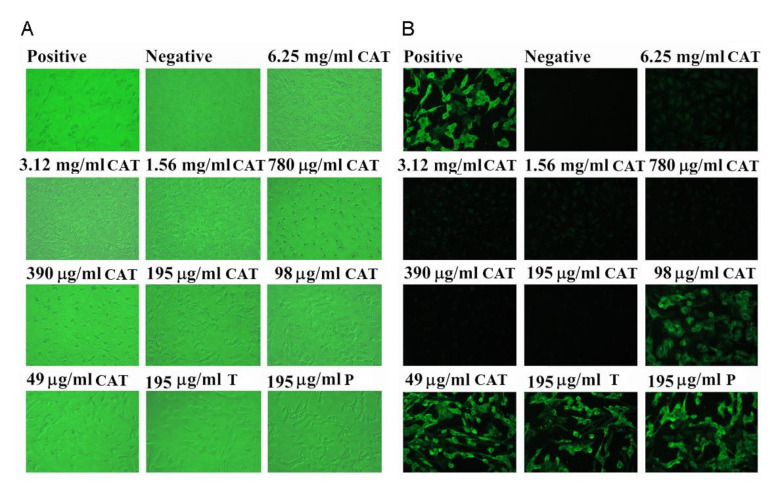
The dose effect of green tea extracts catechins on the morphologic alteration in SARS-CoV-infected Vero E6 cells (**A**). Vero E6 cells were treated with different concentrations of catechins, and the cell morphology was determined at 48 h post-infection. Vero E6 cells were normally expressed in the medium with catechins (6.25 mg–195 μg) treatment, however, the infected Vero E6 cells without catechins treatment died and floated in the medium. The positive panel shows cells infected by SARS-CoV without catechins treatment; while the negative panel shows cells without virus infection. The dose effect of catechins on inhibiting viral antigen synthesis in SARS-CoV-infected Vero E6 cells (**B**). Vero E6 cells were treated with several concentrations of catechins, and an IFA was examined at 48 h post-infection. Viral antigen synthesis was partial blocked at 98 μg/mL of catechins and completely blocked > 195 μg/mL of catechins. The same dose of 195 μg/mL tannin or procyanidin could not inhibit viral synthesis. The positive panel displays the cells infected by SARS-CoV without catechins treatment; whereas the negative panel displays the cells without SARS-CoV infection. T, tannin; P, procyanidin.

Our results showed that catechins inhibited cell damage induced by SARS-CoV and improved the morphology of Vero E6 cells at a concentration of 195 μg/mL and above. A concentration titration experiment revealed that catechins at 195 μg/mL were found to effectively inhibit CPE formation in virus-infected cells. We found that catechins at 195 μg/mL or higher were able to completely inhibited the synthesis of viral antigens in this immunoblot analysis (Figure 2A). As shown in Figure 2B, the expression of viral antigen was inhibited by catechins in a dose-dependent manner. Each virus-infected cell without extract treatment emitted a bright fluorescent light. At concentrations of 195 μg/mL and higher, catechins completely inhibited the viral antigen synthesis. Therefore, according to our data, the effective concentration of catechins to inhibit 50% of viral antigen synthesis was estimated to be within the range of 100 to 200 μg/mL. This experiment was performed in triplicate and the representative results were demonstrated. The concentration of catechins that decreased cell viability to 50% as the cellular toxicity of catechins, was approximately 25 mg after 48 h of catechins treatment.

**Figure 3:**
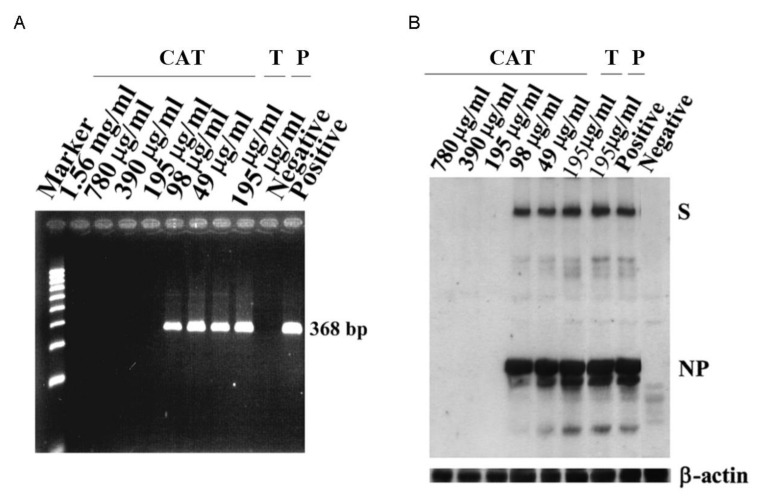
The effect of catechins on SARS-CoV antigen synthesis in Vero E6 cells. The effect of catechins on decreasing the viral yields in the culture supernatants of SARS-CoV-infected Vero E6 cells (**A**). Viral RNA was obtained from culture supernatants of SARS-CoV-infected Vero E6 cells and analyzed by RT-PCR. PCR products of 368 bp were observed when Vero E6 was treated with catechins at 98 μg/mL and lower concentration whereas no PCR products were detected when cells were treated with catechins at higher concentrations (>195 μg/mL). The original immunoblot displays that catechins, at a concentration of 195 μg/mL or higher, completely inhibited the synthesis of viral antigens of SARS-CoV in Vero E6 cells (**B**). The viral antigens spikes protein (S) and nucleocapsid protein (NP) are identified. The bottom panel is an immunoblot stained with anti-β-actin antibody as an internal control. It is clearly indicated that catechins inhibited SARS-CoV budding in a dose-dependent manner. The same dose of 195 μg/mL tannin or procyanidin could not inhibit viral synthesis. Representative results from five separate experiments are shown. Positive, cells infected by SARS-CoV without catechins treatment; negative, cells without virus infection. T, tannin; P, procyanidin.

There was no specific PCR product of 368 bp identified in the medium of SARS-CoV infected cells when catechins were administered at 195 μg/mL or higher concentrations (Figure 3A). The band of the corresponding length was found in the PCR-amplified product from the medium of Vero E6 cells treated with 98 μg/mL catechins. Obviously, catechins exerted a dose-dependent inhibition on virus synthesis in the infected cells. PCR products of 368 bp were found when Vero E6 was treated with catechins at 98 μg/mL and lower concentration. No PCR products were detected when cells were treated with catechins at higher concentrations (>195 μg/mL). Our data clearly demonstrated that catechins inhibited SARS-CoV budding in a dose-dependent manner. Representative results of the immunoblots from spike proteins (S) and nucleocapsid protein (NP) indicated that catechins at 195 μg/mL and higher concentrations (>195 μg/mL) inhibited S and NP expression (Figure 3B). 

### 3.1. Catechins Decreased CD4^+^ T Lymphocytes and Increased CD8^+^ T Lymphocytes Number 

**Figure 4:**
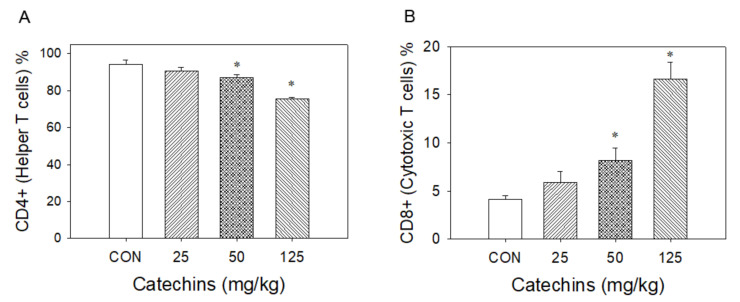
After 4 weeks of catechins ingestion, the percentage change of splenic T lymphocytes in mice. Using flow cytometry assay, catechins treatment significantly and dose-dependently decreased % of splenic CD4^+^ T lymphocytes (**A**) and increased % of CD8^+^ T lymphocytes (**B**); * *p* < 0.05 vs. control (0 mg/kg).

**Figure 5:**
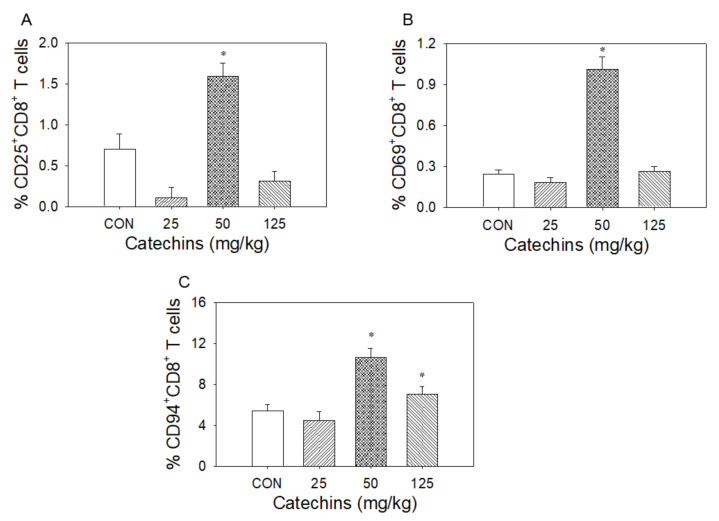
Effect of four weeks of dietary catechins on CD25/CD69/CD94-NKG2A signaling in mice splenic CD8^+^ T lymphocytes. After 4 weeks of catechins ingestion, the percentage change of CD25 (**A**), CD69 (**B**) and CD94-NKG2A (**C**) splenic CD8^+^ T lymphocytes is noted in mice. Using flow cytometry assay, catechins treatment significantly increased percentage of CD25, CD69, and CD94-NKG2A receptor expression in splenic CD8^+^ T lymphocytes primarily at the dosage of 50 mg/kg; * *p* < 0.05 vs. control (0 mg/kg).

Using flow cytometric analysis, four-week catechins ingestion (from 25 to 125 mg/kg) dose-dependently decreased CD4^+^ helper T-cell percentage (from 94 to 75%) and increased CD8^+^ (cytotoxic) T-cell percentage (from 4.1 to 16.5%) (Figure 4). At the dose of 50 mg/kg, catechins upregulated CD25^+^ (from 0.70 to 1.59%), CD69^+^ (from 0.24 to 1.01%), and CD94-NKG2A (from 5.5 to 10.6%) expression in CD8^+^ T lymphocytes (Figure 5).

### 3.2. Type I Cytokines in mRNA and Protein Levels Were Enhanced in Catechins-Treated Mice

**Figure 6:**
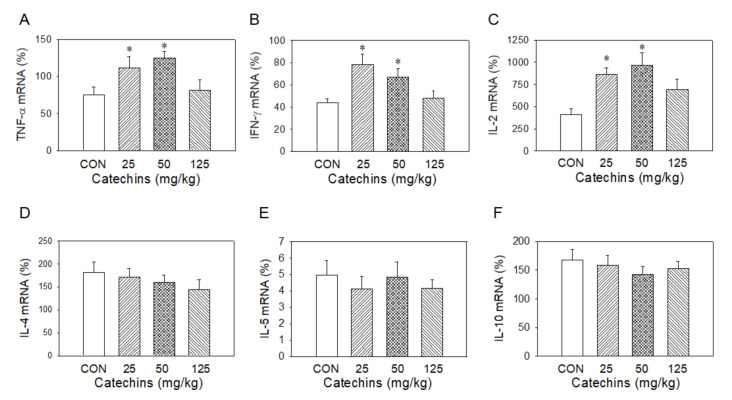
Four-week catechins ingestion significantly increased splenic mRNA expression of type I cytokine TNF-α (**A**), IFN-γ (**B**) and IL-2 (**C**), but had no effect on type II cytokine mRNA IL-4 (**D**), IL-5 (**E**), and IL-10 (**F**); * *p* < 0.05 vs. control group (0 mg/kg).

**Figure 7:**
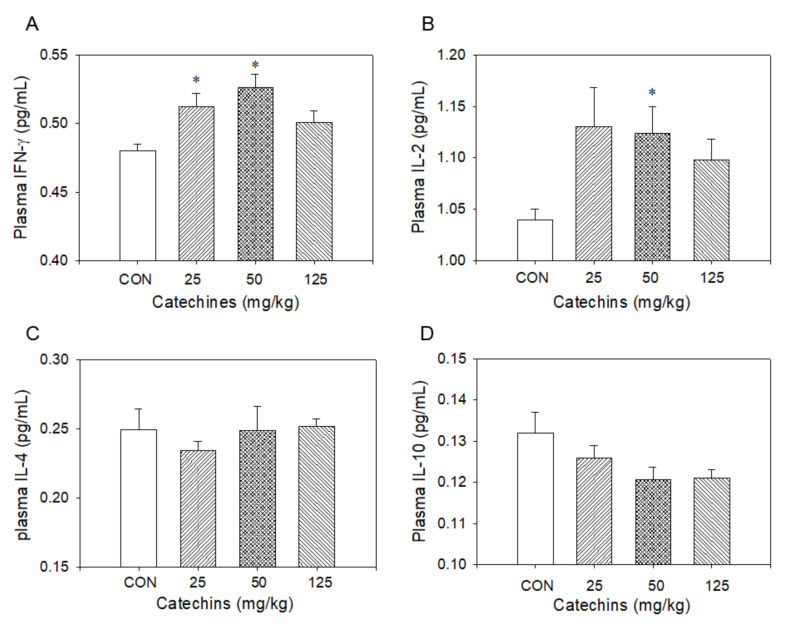
Four-week catechins ingestion especially at dosage of 50 mg/kg increased plasma type I cytokine INF-γ (**A**) and IL-2 (**B**), not type II cytokine IL-4 (**C**) and IL-10 (**D**) level, by using an ELISA assay; * *p* < 0.05 vs. control (0 mg/kg) group.

Type-I T cells produced type-I cytokines, such as IL-2, TNF-α, and IFN-γ, to enhance cellular response to remove infected cells, whereas Type-II T cells produced type II cytokines, such as IL-4, IL-5, and IL-10, to increase antibodies to attack exogenous antigen to prevent host-cell infection [21]. Wondering whether catechins affected immune responses or not, we determined cytokine production in mRNA and protein levels after 4-week catechins ingestion. T cells presented in splenocytes were activated by 10 μg/mL ConA for 3 days and the total RNA was extracted for the quantitative PCR to evaluate type I (TNF-α, IL-2, and IFN-γ) and type II (IL-4, IL-5, and IL-10) cytokine expression. The expression was calibrated with internal control-TATA box binding protein. We found catechins administration selectively induced type I mRNA cytokines production. On the other hand, it had a minor effect on type II mRNA cytokines induction (Figure 6). After then, we further determined these cytokine expressions via ELISA; there were no differences in protein levels between the two groups without stimulation. We proposed that the phenomenon may be due to no stimuli to these mice. From these observations, we suggest that catechins would selectively enhance type-I immune responses and weaken type-II immune responses simultaneously. To validate the hypothesis, catechins-fed mice were sensitized and challenged by OVA and the changes in cytokine production were evaluated. In response to OVA stimulation, there was increased ratio in type-I/type-II cytokines in the cytokine ELISA assay. We observed that there were mild increases of IFN-γ and IL-2 levels and no change in IL-4 and IL-10 levels in catechins-fed mice (Figure 7).

Four-week catechins-fed mice were immunized and activated as previous description. Conditioned supernatants were collected for further cytokine expression array by cytokine array. From the cytokine array data, we found catechins feeding predominantly affected chemokines expression, including upregulation in CTACK (CCL27), CXCL16, KC, Lymphotactin (XCL1), MCP-1 (CCL1), MCP-5 (CCL12), M-CSF, MIG (CXCL9), MIP-1α (CCL3), and SDF-1α (CXCL12) and downregulation in L-selectin (CD62L), MIP-2, P-selectin (CD62P), RANTES (MCP-2), SCF, TNF, and TNF RI.

### 3.3. Catechins Reduced LPS-Induced ALI and Cytokine Storm

**Figure 8:**
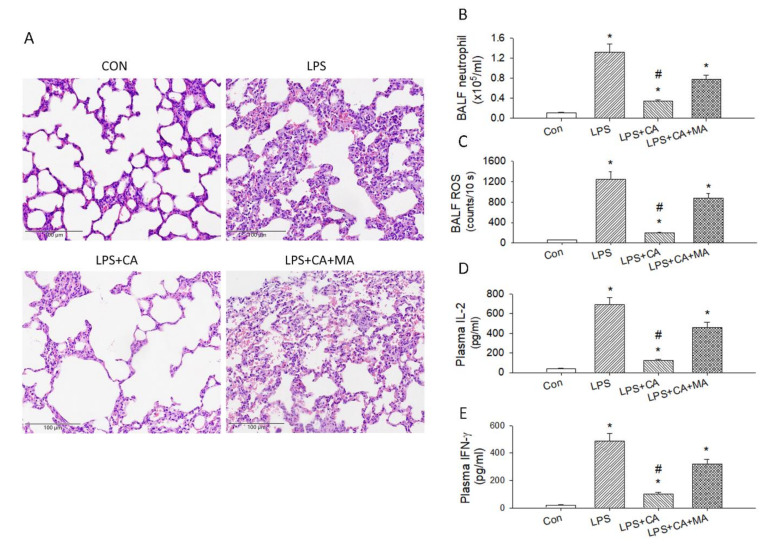
Catechins treatment reduced LPS-induced ALI. (**A**) H&E staining of the lung in the four groups of mice. (**B**) Analysis of neutrophils in the BALF of four groups (*n* = 6). (**C**) The ROS amount in the BALF of these four groups (*n* = 6). The concentration of plasma IL-2 (**D**) and IFN-γ (**E**) of these four groups (*n* = 6); * *p* < 0.05 vs. Con. # *p* < 0.05 vs. LPS.

Because LPS-induced cytokine storm and ALI resembled the conditions evoked by SARS-CoV, we challenged the catechins treated mice with LPS injury. Catechins inhibits ALI through the induction of autophagy in LPS-induced ALI model. Autophagy plays crucial role in regulating inflammatory response by eliminating inflammasome, cytokines, and cellular components, which provides an important mechanism to inhibit inflammation. To explain whether catechins inhibited inflammation of LPS-induced ALI through autophagy activation in vivo, we evaluated the HE staining to evaluate changes of the pulmonary histopathological features. Our results observed that the numbers of inflammatory cell infiltration, vascular congestion and bronchial wall thickening were all increased in LPS-induced ALI model vs. control group (Figure 8A). Importantly, the histological changes markedly reduced after catechins ingestion, and these parameters were reversed with 3-MA treatment. Reactive oxygen species (ROS), IL-2, and IFN-γ are representative proinflammatory factors in many infectious diseases. To further explore the effect of catechins on inflammation of ALI in vivo, we determined the neutrophils and ROS level in the BALF and plasma levels of IL-2 and IFN-γ by ELISA. Our data demonstrated that LPS significantly increased the BALF neutrophils (Figure 8B) and ROS level (Figure 8C) and the production of plasma IL-2 (Figure 8D) and IFN-γ (Figure 8E) (*p* < 0.05). Nevertheless, pretreatment by catechins significantly depressed the levels of neutrophils and ROS in the BALF and plasma levels of IL-2 and IFN-γ (*p* < 0.05), and the inhibitory effects of catechins on these inflammatory factors were reversed by 3-MA in LPS-induced ALI model (*p* < 0.05) (Figure 8B–E). The above results were strongly indicating that catechins decreased lung inflammation of ALI through activation of autophagy in vivo.

### 3.4. Catechins Enhanced Autophagy through the PI3K/AKT/mTOR Pathway in LPS-ALI

**Figure 9:**
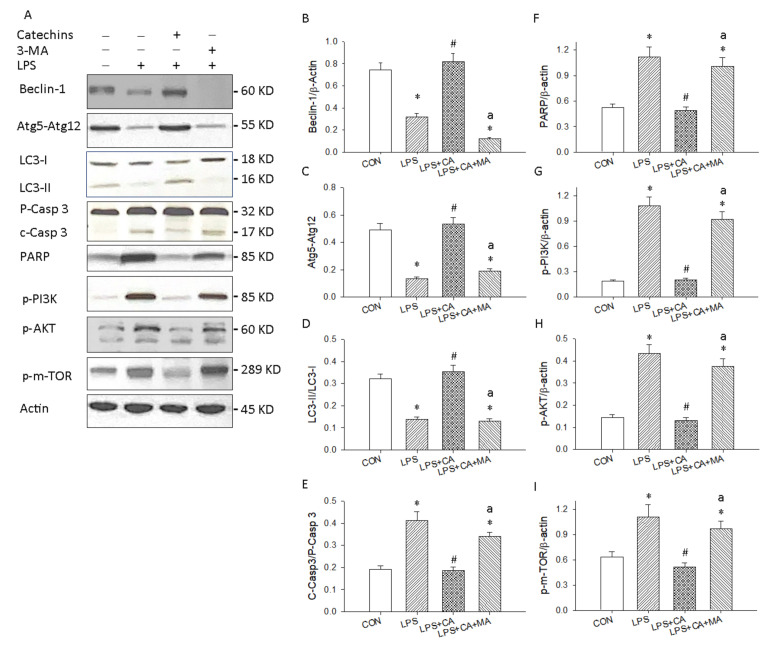
Catechins promoted autophagy dependent protective mechanism against ALI. (**A**) Western blotting detected the levels of autophagy related Beclin-1, Atg5-Atg12 and LC3-II/I, apoptosis related proenzyme and cleavage Caspase 3 and PARP and the PI3K/AKT/m-TOR signaling in lung tissues. Quantitative analysis of autophagy related Beclin-1 (**B**), Atg5-Atg12 (**C**), and LC3-II/I (**D**); apoptosis related C-Casp3/P-Casp3 (**E**); and PARP (**F**), p-PI3K (**G**), p-AKT (**H**), and p-m-TOR (**I**) are displayed in bar graphs, respectively. These experiments were performed three independent times (*n* = 3), and bars represented as mean ± SEM; * *p* < 0.05 vs. CON group; # *p* < 0.05 vs. LPS group; a *p* < 0.05 vs. LPS + CA group.

We examined the effect of catechins on autophagy, apoptosis and PI3K/AKT/m-TOR signaling in LPS-induced ALI by evaluating the protein levels of Beclin-1/Atg5-Atg12/LC3-II, caspase 3/PARP and phosphorylated PIK3/AKT/m-TOR with Western blotting. As shown in Figure 9A of the original Western blot, the expression of Beclin-1 (Figure 9B), Atg5-Atg12 (Figure 9C) and LC3-II (Figure 9D) was significantly depressed in the LPS group vs. control group, whereas cleavage caspase 3 (Figure 9E) and PARP (Figure 9F) were all enhanced in the LPS group vs. control group. Catechins treatment could increase the levels of Beclin-1, Atg5-Atg12, and LC3-II in our LPS-induced ALI model, whereas catechins significantly inhibited cleavage caspase 3/PARP in LPS-induced ALI. Moreover, 3-MA treatment inhibited catechins-activated autophagy-related proteins expression and recovered LPS-enhanced apoptosis signaling. Our results informed that catechins enhanced autophagy-related protective mechanisms in LPS-induced ALI mouse model.

We further determined the PI3K/AKT/mTOR signaling mechanisms on catechins activated autophagy in LPS-induced ALI. Our results demonstrated that the levels of p-PI3K (Figure 9G), p-AKT (Figure 9H), and p-mTOR (Figure 9I) proteins were markedly decreased with catechins treatment (50 mg/kg) and were reversed after 3-MA administration. These results informed that the PI3K/AKT/mTOR pathway involved the enhancement of autophagy by catechins under LPS-induced ALI.

## 4. Discussion

In the present study, we reported the discovery, for the first time, that decaffeinated catechins are able to inhibit SARS-CoV replication at sub-μg/mL concentration. These data are consistent with the recent finding that EGCG inhibits human coronavirus replication in vitro [15]. Our green tea extracts catechins containing six catechins in a defined percentage are available for dietary supplement and have been applied to prevent substance P-induced hyperactive bladder [22], attenuate acute liver injury via inhibiting proinflammatory signaling [23], prevent Helicobacter pylori infection via upregulating autophagy mechanism [24,25], ameliorate cooking-oil-fumes-induced oxidative stress in lung [26], and reduce hemodialysis-enhanced production of oxidative stress and proinflammatory cytokines [18]. A traditional drug or nutritional food component development based on antioxidant, anti-adhesion, and anti-inflammatory catechins as lead compounds represents a more advanced starting point because the safety and clinic experience of this extract have been documented [18,22,23,24,25,26]. Because catechins have been widely used in beverages and food supplements for humans, the findings can be considered for immediate use for prevention and/or treatment of SARS or COVID-19 patients, alone or in combination with other therapies. Determining the best regimen will be performed in humans in carefully controlled trials. Corona virus invades human cells through binding of its distinct surface spike protein (glycoprotein in nature) with a receptor protein (Angiotensin Converting Enzyme 2 (ACE2)) present on the membrane of human cells. The viral spike protein (S protein) and the cognate host-cell receptor ACE2 have been reported as effective and appropriate targets for interventions. The computational study by Jena et al. [14] suggests that catechin, not only exhibit high binding affinity to viral S protein and ACE2 but also to their complex (receptor-binding domain) of the S protein of SARS-CoV-2 and ACE2.

It will be desirable to evaluate the effect of catechins in SARS or COVID-19 animal models or in human clinical trials to confer the efficacy whether catechins may prevent or alleviate SARS injury. Since SARS-CoV was known to replicate actively in intestinal tract [27] and SARS-CoV-2 cell entry factors ACE2 existed in the gastrointestinal tract [28], it is very important to evaluate whether oral catechins can suppress viral replication and reduce the viral load in the lumen of intestine or in the stool. Catechins have a potential to reduce the burden of virus shedding from stool that contaminates the environment and infects others. If this is true, this extract should be considered as a means to prevent the fecal–oral transmission that is speculated as one the major routes of transmission [29]. It is also important to examine whether catechins can be absorbed and passed the first-pass effect to become bioavailable to exert their anti-SARS-CoV activities in lung or lower respiratory tract.

Although several antiviral drugs such as nelfinavir (a HIV-1 protease inhibitor) [30], interferons [31], niclosamine (an existing antihelminthic drug at 1.56 μM) [32] on SARS and intravenous remdesivir and dexamethasone COVID-19 [33] have been endeavored, we would like to emphasize that the tenacious effort to discover novel chemical entities to treat SARS related diseases could also be accompanied by a re-evaluation of existing extracts for unexpected antiviral activities against replication of SARS-CoV.

Infection with SARS-CoV or SARS-CoV-2 may result in severe pneumonia potentially requiring mechanical ventilation and intensive care treatment. A novel ratio of CD8^+^: B cells was significantly lower in intubated versus non-intubated and intubated non-survivors versus survivors [34]. A pathological study of a patient who died from COVID-19 pneumonia displays bilateral diffuse alveolar damage with cellular fibromyxoid exudates, interstitial lymphocyte infiltrates, and multinucleated syncytial cells in the intra-alveolar spaces [8]. Clinical studies suggested that dysregulation of immune response, especially T cells, might be highly involved in the pathological process of COVID-19 [12,35]. Du et al. [36] further indicated that a remarkable reduction in CD8^+^ T cells is one of the predictors for mortality of COVID-19 patients [36]. It is well known that naive CD8^+^ T cells are able to differentiate into a type I cytotoxic T subset that produces IL-2 and IFN-γ, or a type II cytotoxic T subset that produces IL-4, IL-5, and IL-10 [37]. Our data found that catechins ingestion increased the CD8^+^ T-cell percentage, upregulated CD25/CD69/CD94 expression in CD8^+^ T cells and mildly increased type I cytokines release to promote the adaptive immunity against viral infections, such as SARS-CoV or SARS-CoV-2. We suggest that the upregulation of CD25/CD69/CD94 expression by 50 mg/kg of catechins may be a summational effect by the six components including epigallocatechin gallate, epicatechin gallate, epicatechin, epigallocatechin, gallocatechin, and catechin. The respective component of catechins at various dosages may activate or inhibit the CD marker expression by unknown mechanisms. We therefore suggest that increased CD8^+^ T-cell number/ratio and enhanced CD8^+^ T cells’ mediated adaptive immunity may confer anti-SARS virus capability. CD8^+^ T cytotoxic lymphocytes produced type I cytokines, such as IL-2 and IFN-γ, to serve as the first-line defense mechanism and obtained acquired immunity during post-infection or T-cell clone expansion [38]. CD8^+^ T lymphocytes mediate immunosurveillance against persistent virus infections and virus-induced neoplasia and Moser et al. [39] demonstrated that the natural killer cell inhibitory receptor, CD94-NKG2A, is up-regulated by antiviral CD8^+^ T cells during acute polyoma infection and is responsible for down-regulating their antigen-specific cytotoxicity during both viral clearance and virus-induced oncogenesis. CD25 (IL-2 receptor) is highly expressed on functional T lymphocyte membrane and CD25 upregulation promotes functional CD8^+^ T lymphocytes clonal expansion and triggers immune response. CD25 downregulation appears in the human colon cancer, breast cancer, cervical cancer, and renal carcinoma [37]. CD69 functionally drives the clonal expansion in T lymphocyte and natural killer cells‚ lymphocykines secretion, and cytotoxic activity. CD69 activation can be negatively regulated by CD94-NKG2A receptor in order to prevent the overactivation contributing to host-cell damage [40]. Furthermore, high expression of CD94-NKG2 can prevent activation induced apoptotic death in T lymphocytes [41]. In the clinical finding, the high expression of CD94^+^ cells increased the survival rate in the nasal-type extranodal NK/T-cell lymphoma patients [42] or inhibited leukocytes-activation-induced apoptosis [41]. Therefore, a prevention of activation induced CD8^+^ T lymphocyte death and depletion, the upregulation of CD94-NKG2A may increase the sensitivity of T lymphocytes to the antigen, delicately control cytotoxic T lymphocyte response, reduce antigen induced activation of T lymphocyte death, and prolong cytotoxic T lymphocyte reaction, prevent clonal exhaustion and promote the generation of a pool of memory cells [43,44]. Our data from mice in vivo informed us that 4-week catechins treatment dose-dependently decreased the total number of CD4^+^ helper T lymphocytes and increased the total number of CD8^+^ cytotoxic T lymphocytes. The ratio of CD4^+^/CD8^+^ T lymphocytes was significantly decreased after catechins ingestion. In addition, four-week catechins intake significantly increased CD25^+^, CD69^+^ and CD94-NKG2A^+^ expression on CD8^+^ T cytotoxic lymphocytes. According to our data, catechins at dosage of 50 mg/kg displayed the highest expression of CD25/CD69/CD94-NKG2A expression on CD8^+^ T cytotoxic lymphocytes. We suggest that catechins upregulated CD25 and CD69 to activate CD8^+^ T cytotoxic lymphocytes, whereas upregulated CD94-NKG2A can increase the sensitivity of T lymphocytes to the antigen, delicately control cytotoxic T lymphocyte response, reduce antigen induced activation of T lymphocyte death, and prolong cytotoxic T lymphocyte reaction, prevent clonal exhaustion and promote the generation of a pool of memory cells. These data show that catechins treatment, an upregulation in CD25^+^ and CD69^+^ T lymphocytes may implicate a functional response in T cells. The significant elevation of CD25^+^, CD69^+^ and CD94-NKG2A^+^ may impact a negative regulation of enhanced CD25^+^ and CD69^+^ by the upregulation of CD94-NKG2A^+^ T lymphocytes.

In the present study, the reasons for using a LPS model to develop ALI/ARDS can be used as a surrogate for virus induced ALI and cytokine storm as described below. Infections induced by influenza viruses, as well as COVID-19 pandemic induced by SARS-CoV-2 led to ALI and multiple organ failure [9,45], which could occur in LPS induced animal model. SARS-CoV-2 enters the cells through binding to ACE2, a protein highly expressed in the lungs and inhibition of ACE2 could result in the inflammatory bradykinin production in the lung [46]. Furthermore, the inactivation of ACE2 resulting in severe acute pneumonitis was also happened in the LPS induced animal models [46]. On the other hand, dysregulated Toll-like receptor (TLR)-4 activation is involved in LPS-induced acute systemic sepsis, chronic inflammatory diseases, and in viral infections, such as influenza infection. COVID-19 patients upregulate TLR-4-mediated inflammatory signaling that mimics bacterial sepsis [47]. The SARS-CoV-2 induced COVID-19 evoked overexpression of pro-inflammatory cytokines such as IL-6 and TNF-α, which are products of the TLR-4 pathway. The SARS-CoV-2 initially infects cells in the upper respiratory tract and, in some patients, spread very quickly, needing respiratory support and systemically, causing collateral damage in tissues. It has been suggested that overexpression of pro-inflammatory cytokines happens because the SARS-CoV-2 spike protein interacts strongly with TLR-4, causing an intensely exacerbated immune response in the host’s lungs, culminating with the cytokine storm, accumulating secretions and hindering blood oxygenation, along with the immune system attacks the body, leading to multiple organ failure [48]. The small-molecule synthetic TLR-4 antagonist, FP7, protected mice from influenza virus-induced lethality and reduced both proinflammatory cytokine gene expression in the lungs and ALI [49] suggesting viral infection also through activating TLR-4 signaling. SARS-CoV-1 and SARS-CoV-2 having direct and indirect binding to TLR-4, together with other viral precedents, which when combined shed light on the COVID-19 pathophysiological puzzle and the SARS-CoV-2 spike glycoprotein binds TLR-4 and activates TLR-4 signaling to increase cell surface expression of ACE2 facilitating entry [50]. Previous results demonstrated that baicalein inhibited cell damage induced by SARS-CoV-2 and improved the morphology of Vero E6 cells at a concentration of 0.1 μM and above. Baicalein significantly inhibited the body weight loss, the replication of the virus, and relieved the lesions of lung tissue in ACE2 transgenic mice infected with SARS-CoV-2. In addition, in LPS-induced ALI of mice, baicalein improved the respiratory function, inhibited inflammatory cell infiltration in the lung, and decreased the levels of IL-1β and TNF-α in serum, such as the SARS-CoV-2 infection [51]. Based on the above information, we therefore suggest that LPS induced ALI and cytokine storm possibly through ACE2 and TLR-4 mechanisms, such as the SARS-CoV-2 infection. However, the SARS-CoV-2-infected animal model will be performed in the future.

Autophagy has attracted extensive attention in recent years due to the role of autophagy on antiviral capacity to suppress virus infection [11]. The autophagy via several autophagy related proteins can engulf and kill pathogens to protect cells from pathogens and inhibits the secretion of inflammation factors [52]. We therefore challenged the catechins-treated mice with LPS induced cytokine storm and ALI for mimicking SARS-CoV induced injury and determined the possible role of autophagy in LPS-induced ALI. It has been reported EGCG efficiently induced a complete autophagic process to inhibit hepatitis B virus HBV replication [53]. In the present study, we found that LPS could inhibit autophagy in ALI models, and that catechins significantly enhanced the protein levels of Beclin-1 and Atg5-Atg12 and protein ratio of LC3-II/LC3-I and inhibited the C-Caspase 3 and PARP mediated apoptosis implicating catechins enhancing protective autophagy mechanism. Furthermore, we found that catechins promoted the autophagy mechanism through the inhibitory p-PI3K/p-AKT/p-m-TOR signaling pathway. We also evidenced that catechins activated autophagy in LPS-induced ALI and the activation of autophagy by catechins was reversed with 3-MA treatment.

How long could the effect of catechins on CD8^+^ T cells last for? According to our preliminary data by Hsu et al. [18], catechins ingestion increased plasma catechins in EGCG, ECG, etc., approximately 4 h in the healthy human. On the other hand, the time course change of CD11b expression on CD8^+^ T cells induced by EGCG was evaluated previously [54]. Only 5-min exposure to EGCG was enough to cause a marked decrease in the expression of CD11b (17.6% of control), and after 2-h incubation, the impaired CD11b expression was restored to ~37.0%; after 5 h, ~66.5%. We therefore suggest that catechins may affect CD8^+^ T cells for 4–5 h. The dosage of catechins at 195 μg/mL to inhibit SARS CoV replication could be easily reached in the plasma concentration from 50 mg/kg × 50 kg human (2.5 g/day). We have HPLC data that show, in healthy human subjects, that oral intake of catechins tablets at 455 mg can obtain almost 131 μg/mL of catechins concentration in the plasma. Total 2.5 g/day dosage can be separated into twice (1.25 g × 2) or three times (0.83 g × 3) per day and obtained the plasma catechins concentration at 239 μg/mL and 359 μg/mL, respectively. These two concentrations of plasma catechins should inhibit SARS CoV replication in the human subjects.

## 5. Conclusions

**Figure 10:**
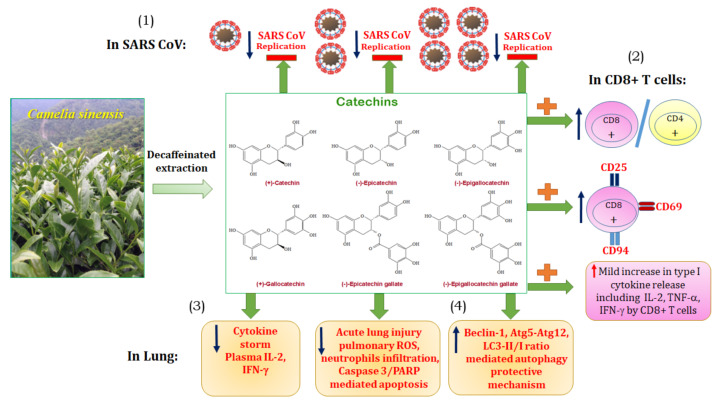
The summary diagram is demonstrated. It is suggested that catechins could be an available candidate drug to meet the requirements, including (**1**) the direct inhibiting SARS-CoV replication, (**2**) the mild enhancement of CD8^+^ mediated adaptive immunity, (**3**) the decrease of cytokine storm, and (**4**) the promoting autophagy-dependent protective mechanism to ameliorate acute lung injury.

In summary, as shown in Figure 10, we suggest that catechins could be available candidate drugs to meet the requirements, including (1) the direct inhibiting SARS-CoV replication, (2) the enhancement of CD8^+^ mediated adaptive immunity, (3) the decrease of cytokine storm, and (4) the promoting autophagy-dependent protective mechanism to ameliorate ALI.

## Data Availability

The data that support the findings of this study are available from the corresponding author upon reasonable request.

## Abbreviations

ALI	acute lung injury
ARDS	acute respiratory distress syndrome
BALF	bronchoalveolar lavage
C	catechin
CAF	caffeine
CG	catechin gallate
Con A	concanavalin A
COVID-19	coronavirus disease 2019
CPE	cytopathic effects
EC	epicatechin
ECG	epicatechin gallate
EGC	epigallocatechin
EGCG	epigallocatechin gallate
GC	gallocatechin
IFA	immunofluorescence assay
LPS	lipopolysaccharide
3-MA	3-methyladenine
MERS	middle east respiratory syndrome
MTS	3-(4, 5-dimethylthiazol-2-yl)-5-(3-carboxymethoxyphenyl)-2-(4-sulfophenyl)-2H-tetrazolium
OVA	ovalbumin/alum
ROS	reactive oxygen species
SARS	severe acute respiratory syndrome
SARS-CoV	severe acute respiratory syndrome-associated coronavirus
